# The 30-year evolution of airway pressure release ventilation (APRV)

**DOI:** 10.1186/s40635-016-0085-2

**Published:** 2016-05-20

**Authors:** Sumeet V. Jain, Michaela Kollisch-Singule, Benjamin Sadowitz, Luke Dombert, Josh Satalin, Penny Andrews, Louis A. Gatto, Gary F. Nieman, Nader M. Habashi

**Affiliations:** Department of Surgery, SUNY Upstate Medical University, 750 E Adams St, Syracuse, NY 13210 USA; Multi-trauma Critical Care, R Adams Cowley Shock Trauma Center, University of Maryland Medical Center, 22 South Greene Street, Baltimore, MD USA; Department of Biological Sciences, 10 SUNY Cortland, Cortland, NY 13045 USA

**Keywords:** APRV, Ventilator-induced lung injury, ARDS, Lung protection

## Abstract

Airway pressure release ventilation (APRV) was first described in 1987 and defined as continuous positive airway pressure (CPAP) with a brief release while allowing the patient to spontaneously breathe throughout the respiratory cycle. The current understanding of the optimal strategy to minimize ventilator-induced lung injury is to “open the lung and keep it open”. APRV should be ideal for this strategy with the prolonged CPAP duration recruiting the lung and the minimal release duration preventing lung collapse. However, APRV is inconsistently defined with significant variation in the settings used in experimental studies and in clinical practice. The goal of this review was to analyze the published literature and determine APRV efficacy as a lung-protective strategy. We reviewed all original articles in which the authors stated that APRV was used. The primary analysis was to correlate APRV settings with physiologic and clinical outcomes. Results showed that there was tremendous variation in settings that were all defined as APRV, particularly CPAP and release phase duration and the parameters used to guide these settings. Thus, it was impossible to assess efficacy of a single strategy since almost none of the APRV settings were identical. Therefore, we divided all APRV studies divided into two basic categories: (1) fixed-setting APRV (F-APRV) in which the release phase is set and left constant; and (2) personalized-APRV (P-APRV) in which the release phase is set based on changes in lung mechanics using the slope of the expiratory flow curve. Results showed that in no study was there a statistically significant worse outcome with APRV, regardless of the settings (F-ARPV or P-APRV). Multiple studies demonstrated that P-APRV stabilizes alveoli and reduces the incidence of acute respiratory distress syndrome (ARDS) in clinically relevant animal models and in trauma patients. In conclusion, over the 30 years since the mode’s inception there have been no strict criteria in defining a mechanical breath as being APRV. P-APRV has shown great promise as a highly lung-protective ventilation strategy.

## Review

### Introduction

**Fig. 1:**
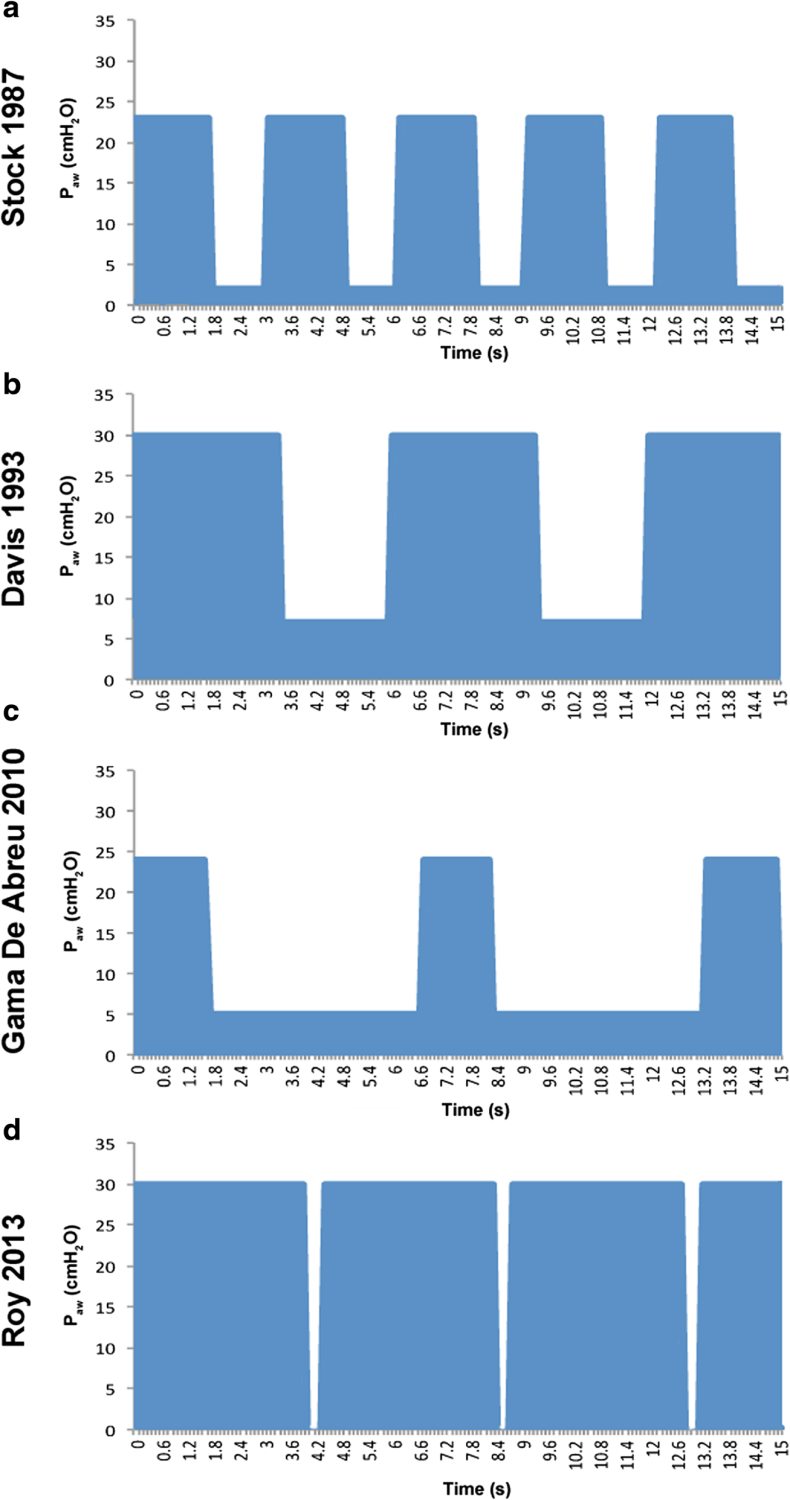
Comparison of APRV pressure waveforms. Artistic depiction of airway pressure waveforms, all of which were defined as APRV, illustrating the significant variability in what has been defined as an APRV breath. Stock in 1987 used 60 % CPAP with T_Low_ of 1.27 s and a respiratory rate (RR) of 20 [2]. Davis in 1993 used a similar %CPAP, but decreased the RR by prolonging T_High_ and T_Low_ [3]. Gama de Abreau in 2010 simulated conventional ventilation with a prolonged T_Low_ and short T_High_ [4]. Finally, Roy in 2013 used a very brief adaptive T_Low_ and large T_High_ with 90 % CPAP [5]. Of note, though the ventilator pressure is set at zero, this does not reflect true pressure as the brief T_Low_ prevents full deflation of the lung, and thus prevents end-expiratory pressure from reaching zero. Figures **a**–**c** are examples of fixed-APRV (F-APRV) and figure **d** of personalized APRV (P-APRV)

Stock and Downs first defined airway pressure release ventilation (APRV) as maintenance of a continuous positive airway pressure (CPAP) that allows for spontaneous breaths without significant airway pressure fluctuation and a brief cyclic release phase for efficient ventilation (i.e., CPAP with release) [1]. However, the flexibility of this definition has become an Achilles heel of sorts since a wide variety of APRV settings have all been used in the literature making comparison between studies impossible. Therefore, the current acronym “APRV” is a nebulous term identifying a ventilator mode without a precisely defined mechanical breath structure or ventilator settings. Variability in settings, and thus mechanical breath structure, significantly changes how the lung “sees” the breath being delivered. The first description of APRV published by Stock et al. in 1987 was, “APRV is a new way to administer simultaneously a supportive level of CPAP and assist CO_2_ elimination” [2]. Thus, the original definition of APRV was simply CPAP with a release to eliminate CO_2_. CPAP with a release is a very general description of a mechanical breath and can define all of the mechanical breaths seen in Fig. 1. Indeed, all of these mechanical breaths were defined as APRV [2–5]. As can be readily seen, the biggest difference between these APRV breaths is the duration at inspiration and expiration. Since the original definition of APRV was CPAP with a release, which did not specify the duration of the CPAP or release time, all of these breaths fit the original definition. Over the 30 years since APRV was originally described, many combinations of CPAP and release times have been used and all defined as APRV.

In this review, we examine published original research in both animal and human trials in which the authors stated they utilized APRV, or a comparable but differently named ventilator mode. Our goals with this review were as follows: (1) to examine the historical evolution of APRV methodology; (2) to evaluate the efficacy of the APRV methodologies versus conventional positive pressure ventilation (CPPV); and (3) to determine if there are optimal APRV settings for maximal lung protection.

The evolution of APRV mostly revolves around modifying the CPAP and release time durations (time at expiration—T_Low_) (Tables 1, 2, 3, and 4, %CPAP and T_Low_). However, the most significant evolution in APRV has been the development of the ability to personalize the expiratory duration to precisely meet the needs of the patient’s changing lung physiology. The advantage of this method is that expiratory duration is set to maintain and open and stable the lung, regardless the level of lung pathology. Since there was no consistency in the CPAP and release time duration in the published literature, we chose to separate APRV strategies into two categories: fixed- (F-APRV) and personalized (P-APRV) APRV. If the expiratory duration or release time was not adjusted by mechanical changes in the lung, regardless of duration of CPAP or release time, it was categorized as F-APRV. If the APRV strategy sets expiratory duration by changes in lung mechanics, using the slope of the expiratory flow curve, it was categorized as P-APRV (Fig. 1).

**Table 1:** Summary of animal studies utilizing F-APRV

First author	Year	*n*	Animal	Study design	% CPAP	T_Low_	Findings
Stock [2]	1987	10	Mongrel dog	Crossover CPPV vs. APRV	58 %	1.27 s	APRV improved oxygenation with lower PIP and without cardiopulmonary compromise
Rasanen [21]	1988	10	Mongrel dog	Crossover CPPV vs. CPAP vs. APRV	50 %	1.5 s	CPPV impaired circulatory function and tissue oxygen balance, APRV had higher systemic vascular resistance and decreased pulmonary vascular resistance
Martin [17]	1991	7	Neonatal sheep	Crossover Spont vs. CPAP vs. CPPV vs. APRV	50 %	1 s	APRV augmented alveolar ventilation vs. CPAP, and had lower Paw than PPV without compromised cardiovascular function
Smith [23]	1995	5	Swine	Crossover CPAP vs. APRV	80 %	1.1 s exp flow 0	APRV maintains oxygenation without hemodynamic compromise
Neumann [19]	2001	9	Swine	Crossover CPAP vs. APRV +/− PEEP	67 %	1 s	APRV decreased O2 compared with CPAP, No difference with PEEP
Hering [13]	2003	12	Swine	Crossover APRV +/− SB	50 %	N/A	APRV + SB increased oxygenation and cardiovascular function
Wrigge [24]	2003	24	Swine	Randomized prospective APRV +/− SB	50 %	1.5–2 s	APRV + SB increased oxygenation and cardiovascular function
Neumann [20]	2005	20	Swine	Randomized prospective APRV +/− SB	50 %	1.5 s	APRV + B increased ventilation in dependent lung and decreased shunt
Hering [14]	2005	12	Swine	Crossover APRV vs. SB	50 %	~1.7 s	APRV + SB improved oxygenation after lung injury
Wrigge [25]	2005	22	Swine	Randomized Prospective APRV +/− SB	50 %	1.5–2 s	APRV + SB redistributes ventilation to dependent lung regions and counters cyclic collapse
Hering [12]	2008	12	Swine	Crossover APRV +/− SB	50 %	N/A	APRV + SB improved oxygenation and splanchnic blood flow
Gama de Abreu [9]	2008	12	Swine	Crossover BiPAP + SB, PSV +/− sighs, “noisy” PSV	N/A	exp flow 0	“Noisy” CPPV improved oxygenation by redistributing perfusion
Carvalho [7]	2009	5	Swine	Crossover PSV vs. BiPAP + SB	Titrated by P_aw_	N/A	BiPAP + SB and pressure support had similar oxygenation improvement and did not improve aeration of dependent lung
Gama de Abreu [4]	2010	10	Swine	Crossover PSV vs. BiPAP + SB	25 %	N/A	BiPAP + SB had lower tidal volume with comparable oxygenation and ventilation distribution
Henzler [11]	2010	20	Swine	Randomized prospective APRV +/− SB	42 %	~1.2 s	Elevated IAH impaired respiratory mechanics regardless of SB
Kreyer [16]	2010	12	Swine	Randomized Prospective APRV +/− SB	50 %	1.5–2 s exp flow 0	APRV + SB improved systemic blood flow and cerebrospinal blood flow
Matsuzawa [18]	2010	21	Rabbit	Randomized prospective CPPV vs. LTV vs. APRV	95 %	0.15 s	APRV reduced HMGB1 levels and lung water
Slim [22]	2011	7	Swine	Case series APRV	80 %	N/A	Increased P_aw_ increased pulmonary capillary wedge pressure and left atrial pressure, but these may not correlate with end diastolic volume
Xia [26]	2011	24	Rabbit	Randomized prospective APRV +/− SB	50 %	N/A	APRV + SB improved oxygenation and attenuated VILI
Carvalho [8]	2014	36	Swine	Randomized prospective APRV +/− SB	50 %	N/A	APRV + SB improved oxygenation and reduced lung injury
Guldner [10]	2014	12	Swine	Crossover APRV +/− SB	50 %	~1 s	Higher levels of SB reduce global lung stress and strain with minimal changes in perfusion
Kill [15]	2014	24	Swine	Randomized prospective CPPV vs. Bilevel vs. Compression synchronized ventilation	40 %	3.6 s	CPPV and Bilevel usable during CPR, though compression synchronized ventilation was best

Number of studies: 22

*T*_*Low*_ time at low pressure, *CPPV* conventional positive pressure ventilation, *LTV* low tidal volume ventilation, *CPAP* continuous positive airway pressure, *SB* spontaneous breathing, *PEEP* positive end-expiratory pressure, *PIP* peak inspiratory pressure, *P*_*aw*_ airway pressure, *PSV* pressure support ventilation, *BiPAP* biphasic positive airway pressure

**Table 2:** Summary of animal studies utilizing P-APRV

First author	Year	*n*	Animal	Study design	%CPAP	T_Low_	Findings
Albert [49]	2011	22	Swine	Randomized prospective, CPPV vs. LTV vs. APRV vs. HFOV	90 %	50–75 % PEF	APRV increased oxygenation and ventilation with reduced cytokines compared to LTV
Roy [57]	2012	8	Swine	Randomized prospective, CPPV vs. APRV	90 %	75 % PEF	Early APRV prevented ARDS with improved oxygenation, histopathology, and surfactant protein preservation
Emr [62]	2013	16	Rat	Randomized prospective, spont vs. CPPV vs. APRV	90 %	75 % PEF	Early APRV prevented ARDS with improved oxygenation and histopathology
Roy [5]	2013	12	Swine	Randomized prospective, sham vs. LTV vs. APRV	90 %	75 % PEF	Early APRV prevented ARDS with improved oxygenation and histopathology with reduced inflammatory markers
Roy [61]	2013	9	Rat	Randomized prospective, CPPV vs. APRV	90 %	75 % PEF	Early APRV prevented ARDS with improved oxygenation and histopathology
Kollisch-Singule [55]	2014	8	Rat	Randomized prospective, CPPV vs. APRV	90 %	10 or 75 % PEF	APRV with low expiratory time reduced conducting airway microstrain
Kollisch-Singule [54]	2014	6	Rat	Randomized prospective, CPPV with PEEP vs. APRV	90 %	10 or 75 % PEF	APRV with low expiratory time reduced alveolar microstrain
Davies [52]	2015	22	Swine	Randomized prospective, LTV vs. APRV	90 %	75 % PEF	APRV increased oxygenation compared with LTV, APRV had a “trend towards” increased cerebral ischemia.
Arrindell [51]	2015	19	Preterm swine	Randomized prospective, CPPV vs. APRV	N/A	75 % PEF	APRV increased oxygenation without change in histopathology or inflammatory markers
Kollisch-Singule [56]	2015	14	Rat	Randomized prospective, uninjured vs. LTV vs. APRV	90 %	75 % PEF	APRV approximated control lungs best with increased homogeneity compared to LTV. LTV with high PEEP reduced heterogeneity.

Number of studies: 10

*T*_*Low*_ time at low pressure, *CPPV* conventional positive pressure ventilation, *LTV* low tidal volume ventilation, *CPAP* continuous positive airway pressure, *PEF* peak expiratory flow, *SB* spontaneous breathing, *PEEP* positive end-expiratory pressure, *PIP* peak inspiratory pressure

**Table 3:** Summary of human trials using F-APRV

First author	Year	*n*	Study design	%CPAP	T_Low_	Findings
Garner [31]	1988	14	Crossover CPPV baseline with APRV wean	N/A	1.5 s	APRV maintained similar oxygenation with >50 % reduced PIP
Rasanen [40]	1991	50	Crossover PEEP titrated CPPV vs. APRV	50 %	1.5 s	APRV maintained similar oxygenation with >50 % reduced PIP
Cane [28]	1991	18	Crossover CPPV vs. APRV	67 %	1.5 s	APRV maintained similar oxygenation and cardiopulmonary function with reduced PIP
Davis [3]	1993	15	Crossover CPPV vs. APRV	32 %	2.6 ± 0.6	APRV maintained similar oxygenation with >50 % reduced PIP and reduced PEEP
Chiang [29]	1994	18	Crossover CPPV vs. APRV	66 %	1.5 s	APRV maintained similar oxygenation with >50 % reduced PIP
Sydow [42]	1994	18	Crossover CPPV vs. APRV	80 %	0.5–0.7	APRV maintained similar oxygenation with decreased A-a gradient after 8 h and reduced PIP
Bratzke [27]	1998	20	Crossover CPPV vs. APRV	88 %	1	APRV maintained similar oxygenation with reduced PIP
Kaplan [36]	2001	12	Crossover Inverse ratio PPV vs. APRV	85 %	0.8	APRV is safe, decreases PIP and need for sedation/paralytics/pressors, increases CI
Putensen [39]	2001	30	Randomized prospective CPPV vs. APRV	Identical to CPPV	Exp flow 0	APRV + SB maintained increased oxygenation, CI, and pulmonary compliance with reduced ALI/ARDS incidence and sedative requirements
Schultz [41]	2001	15	Crossover CPPV vs. APRV	N/A	N/A	APRV maintained similar oxygenation with >50 % reduced PIP
Wrigge [45]	2001	14	Randomized prospective APRV +/− automatic tube compensation	N/A	N/A	APRV with tube compensation increased end-expiratory lung volume and minute ventilation without affecting oxygenation or cardiopulmonary status
Hering [34]	2002	12	Crossover APRV +/− SB	N/A	N/A	APRV + SB had increased renal blood flow and glomerular filtration rate
Varpula [43]	2003	33	Randomized prospective CPPV vs. APRV	N/A	Exp flow 0	APRV feasible in prone positioning and increased oxygenation at 24 h
Varpula [44]	2004	58	Randomized prospective CPPV vs. APRV	80 %	1	APRV had similar mortality and ventilator free days
Dart [30]	2005	46	Crossover CPPV vs. APRV	N/A	40–50 % PEF	APRV reduced PIP and increased oxygenation
Liu [37]	2009	58	Retrospective case-control CPPV vs. APRV	67 %	~1.5	APRV reduced pressor use/A-a gradient and increased oxygenation
Kamath [35]	2010	11	Retrospective cohort CPPV vs. APRV	70 %	1.2 ± 0.9	APRV had no adverse effects on blood pressure or urine output
Gonzalez [32]	2010	468	Case matched retrospective CPPV vs. APRV	70 %	N/A	APRV maintained similar oxygenation with reduced PIP and associated increased tracheostomy rate
Maxwell [38]	2010	63	Randomized prospective LTV vs. APRV	N/A	25–75 % PEF	APRV had similar physiological parameters despite increased disease severity at baseline
Hanna [33]	2011	45	Retrospective case series CPPV vs. APRV	N/A	N/A	APRV had increased P/F Ratio, lung procurement rate with similar graft survival rate
Maung [46]	2012	38	Retrospective case series APRV	85 %	0.8–1	Switching from CPPV to APRV improved oxygenation and decreased PCO_2_ without hemodynamic compromise
Maung [47]	2012	362	Retrospective case series CPPV vs. APRV	N/A	N/A	APRV had increased ventilator days.
Testerman [48]	2013	48	Case-matched retrospective APRV; obese vs. nonobese	N/A	N/A	APRV in morbidly obese similar to nonobese, though morbidly obese required extended care after discharge more often

Number of studies: 23

*T*_*Low*_ time at low pressure, *CPPV* conventional positive pressure ventilation, *LTV* low tidal volume ventilation, *CPAP* continuous positive airway pressure, *PEF* peak expiratory flow, *SB* spontaneous breathing, *PEEP* positive end-expiratory pressure, *PIP* peak inspiratory pressure

**Table 4:** Summary of human trial utilizing P-APRV

First author	Year	*n*	Study design	%CPAP	T_Low_	Findings
Yoshida [60]	2009	18	Retrospective case-control LTV vs. APRV	N/A	50–75 % PEF	APRV + SB had increased oxygenation and MAP and decreased atelectasis
Walsh [58]	2011	20	Retrospective case series CPPV vs. APRV	>80 %	50–75 % PEF	APRV improved pulmonary blood flow after tetralogy of fallot repair or cavopulmonary shunt
Andrews [50]	2013	66,099	Retrospective review CPPV vs. early APRV	90 %	75 % PEF	Early APRV decreased ARDS incidence tenfold and mortality threefold
Kawaguchi [53]	2014	13	Retrospective case series CPPV vs. APRV	90 %	50–75 % PEF	APRV safe in pediatric ARDS without hemodynamic compromise
Yehya [59]	2014	104	Retrospective cohort HFOV vs. APRV	N/A	50–75 % PEF	APRV had no mortality effect compared to oscillatory ventilation as rescue treatments

Number of studies: 5

*T*_*Low*_ time at low pressure, *CPPV* conventional positive pressure ventilation, *LTV* low tidal volume ventilation, *PEF* peak expiratory flow, *SB* spontaneous breathing

### Methodology

A PubMed search of the terms “Airway Pressure Release Ventilation”, “APRV”, “Bi-Vent”, “APRV/Biphasic” and “Bi-Level” and “Mechanical Ventilation” OR “Ventilator” was conducted alone or in combination. English language studies between the years 1987 and 2015 were included, and studies testing non-invasive ventilator strategies alone, reviews, editorials, and case studies were excluded, yielding 52 articles. Of the 60 articles, 32 were animal studies and 28 human studies (Tables 1, 2, 3, and 4). APRV methodologies were analyzed by examining the following settings: (1) pressure during inspiration/CPAP phase (P_High_); (2) time during inspiration/CPAP phase (T_High_); (3) pressure during expiration/release phase (P_Low_); (4) time during expiration/release phase (T_Low_); and (5) percent CPAP (%CPAP) to reflect the time spent at P_High_ relative to the entire breath duration $\left(\frac{T_{\mathrm{High}}}{T_{\mathrm{High}}+{T}_{\mathrm{Low}}}\times \kern0.5em 100\right)$. In addition, we assessed the factors used to titrate these settings including respiratory rate or partial pressure of carbon dioxide (PCO_2_) for T_High_ and T_Low_ and oxygenation or plateau pressure for P_High_.

**Fig. 2:**
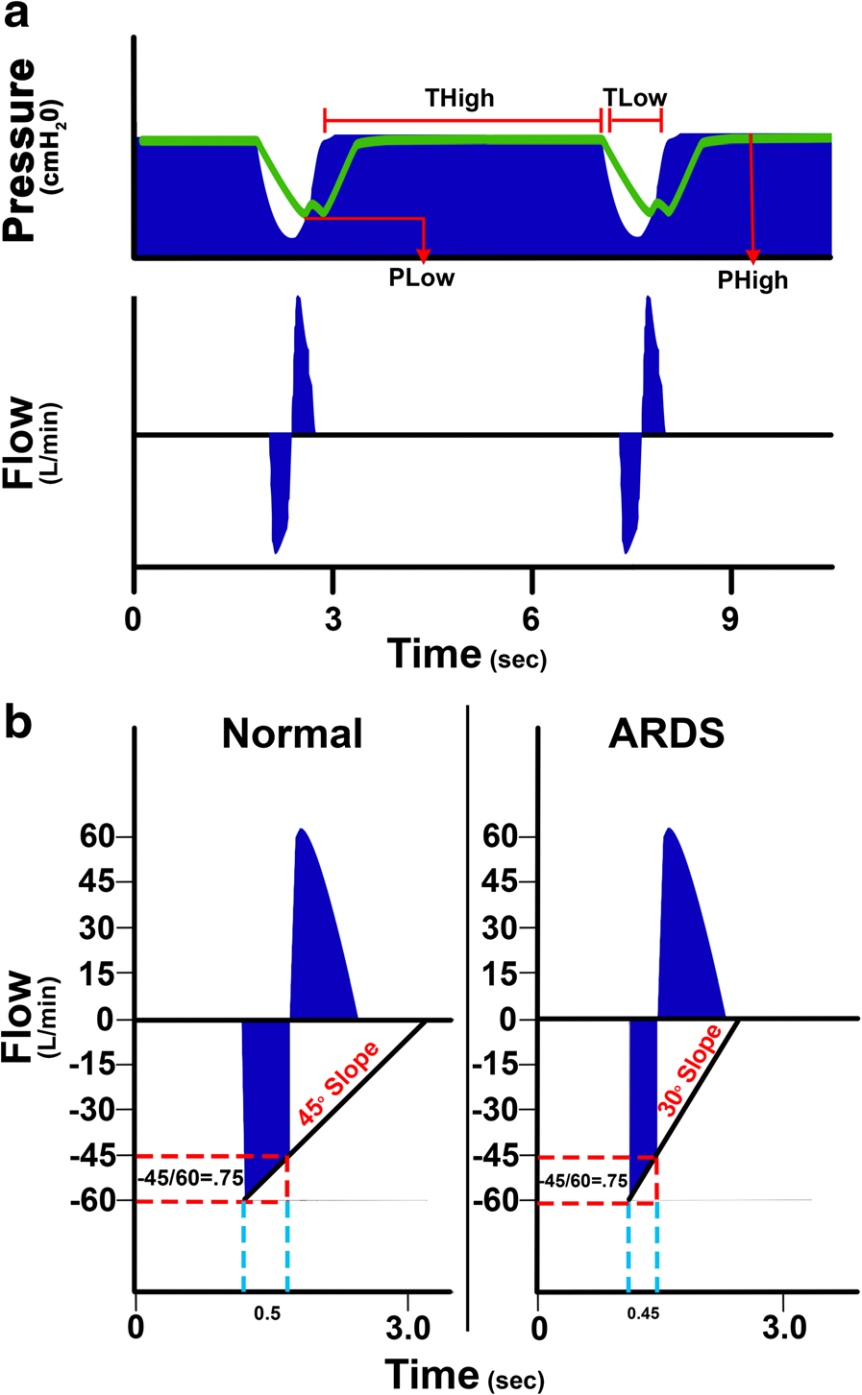
Method of setting expiratory duration (T_Low_). **a** Typical personalized airway pressure release ventilation (P-APRV) airway pressure and flow curves. Correctly set P-APRV has a very brief release phase (time at low pressure—T_Low_) and CPAP phase (time at high pressure—T_High_) [6]. The T_High_ is ~90 % of each breath. The two other P-ARPV settings are the pressure at inspiration (P_High_) and at expiration (P_Low_). T_Low_ is sufficiently brief such that end-expiratory pressure (P_Low_) never reaches 0 cmH_2_O measured by the tracheal pressure (*green line*). **b** Maintain alveolar stability by adaptively adjusting the expiratory duration as directed by the expiratory flow curve. The rate of lung collapse is seen in the normal (slope 45°) and acutely injured lung (ARDS, slope 30°). ARDS causes a more rapid lung collapse due to decreased lung compliance. Our preliminary studies have shown that if the end-expiratory flow (EEF; −45 L/min) to the peak expiratory flow (PEF; −60 L/min) ratio is equal to 0.75, the resultant T_Low_ (0.5 s) is sufficient to stabilize alveoli [54, 55]. The lung with ARDS collapses more rapidly such that the EEF/PEF ratio of 75 % identifies an expiratory duration of 0.45 s as necessary to stabilize alveoli. Thus, this method of setting expiratory duration is adaptive to changes in lung pathophysiology and personalizes the mechanical breath to each individual patient

Reviewing the evolution of APRV from its inception in 1987, we noted a major paradigm shift in the way APRV is set. Initially, the variations in settings were to the inspiratory and expiratory duration time that were fixed and not adjusted to changes in lung mechanics (Fig. 1a–c). In 2005, Habashi published a paper with a novel method of setting the expiratory duration based on changing lung mechanics identified by the slope of the expiratory flow curve (Figs. 1d and 2) [6]. This novel method of setting expiratory duration sets this personalized APRV (P-APRV) strategy (Figs. 1d and 2) apart from all other fixed setting-APRV (F-APRV) strategies.

Thus, we chose to divide our review of APRV efficacy into these two basic categories. The F-APRV breath with a relatively short inspiratory time (T_High_) occupying <90 % of total cycle time (Fig. 1a–c) of each breath with a fixed expiratory time (T_Low_) that is not adjusted based on changing lung mechanics. The second category originally described by Habashi [6] was a P-APRV breath with the following settings: (1) the inspiratory pressure (P_High_) is set to the desired plateau pressure,; (2) the T_High_ is typically set to occupy 90 % of the total cycle time of each breath (Fig. 2a); (3) the T_Low_ set based on changes in lung mechanics by analyzing the slope of the expiratory flow curve (Fig. 2b); and (4) the expiratory pressure (P_Low_) is set at 0 cmH_2_O to minimize resistance to convective expiratory gas flow and maximize ventilation. The short duration at end expiration prevents the airway pressure from reaching atmospheric pressure, thus maintaining a positive end-expiratory pressure. Based on the above criteria, the studies reviewed were placed into either the F-APRV (Tables 1 and 3) or P-APRV (Tables 2 and 4) category.

### Results

Animal (Tables 1 and 2) and human (Tables 3 and 4) studies were examined for APRV settings and efficacy. As described above, the APRV methodologies were subdivided into two categories: F-APRV; (Tables 1 and 3) and P-APRV (Tables 2 and 4). The majority of the animal studies (69 % of total) [2, 4, 7–26] and human studies (82 % of total) [3, 27–48] were in the F-APRV category.

#### How expiratory duration was personalized

The expiratory flow curve is analyzed, and the ratio of the end-expiratory flow (EEF) to the peak expiratory flow (PEF) is set so that the EEF/PEF ratio is 75 % [5, 49–62] (Fig. 2b) based on the methodology described by Habashi [6] (Tables 2 and 4). The T_Low_ is thus personalized based on alterations in lung mechanics, identified by changes in the slope of the expiratory flow curve (Fig. 2b). Using EEF/PEF ratio of 75 % results in a brief T_Low_ ranging from 0.3 to 0.6 s (Fig. 1d). However, recent animal experiments have shown the optimal EEF/PEF ratio necessary to open and stabilize the lung is 75 % [54–56].

#### F-APRV: inspiratory duration

There was considerable variability in the F-APRV settings (i.e., duration at inspiration and expiration) in the papers reviewed, and in multiple papers, the settings were not defined, and thus not included in this review. Swine, dogs, and rabbits were used in the animals studies with the majority of studies using swine (Table 1). In only two of the animal studies were the %CPAP set at ≤80 % of the total cycle time (Table 1) [22, 23]. In one study, %CPAP was set very short at 25 % of the breath cycle time [5]. When both the animal and human studies were analyzed 23/45 studies set %CPAP ≤67 % of the breath and 13/45 had an indeterminate %CPAP (Tables 1 and 3).

#### Expiratory duration

In addition, the T_Low_ in both animal and human studies was prolonged to levels seen in conventional mechanical ventilation (Fig. 1) [3, 9, 15, 16, 24, 25, 43]. Aside from two studies [30, 38], the T_Low_ remained fixed or was titrated based on the PCO_2_ as opposed to being adjusted based on changes in lung mechanics using the expiratory flow curve (Fig. 2). The P_High_ was titrated very differently in the studies reviewed, with a range between 10 cmH_2_O [2, 21, 28, 29, 31] and 35 cmH_2_O [3, 37] depending on whether the target was maximal oxygenation, maximal lung recruitment, or a specific tidal volume. In addition, most of the studies in the F-APRV group set a positive P_Low_ > 0 cmH_2_O [2, 4, 7–11, 13, 14, 16, 18–23, 26, 40].

#### Outcome

The majority of the studies in the F-APRV group were crossover experiments, representing 59 % of the animal studies (Table 1) and 48 % of the human trials (Table 3) [2, 4, 7, 9, 10, 12–14, 17, 19, 21, 23, 32], with the primary goal to demonstrate that APRV could be safely used without adverse effects on lung function or hemodynamic characteristics. Many crossover trials showed similar or increased oxygenation with lower peak pressures and no negative effect on hemodynamics with APRV as compared with CPPV [2, 3, 17, 27–32, 39–42], and some studies showed hemodynamic improvement with APRV [13, 24, 36, 39]. Prospective randomized trials comparing APRV and CPPV showed that APRV is safe and potentially beneficial (Table 3). In none of the studies included in this review did APRV cause a significant negative impact on the physiologic process being studied, and in some prospective randomized studies, APRV was shown beneficial (Tables 1 and 3).

#### P-APRV

Similar to the F-APRV studies, P-APRV either resulted in improved outcomes or no change as compared with CPPV and in none of the studies was APRV shown to be harmful. Unlike the F-APRV studies that were mainly crossover studies, all of the P-APRV animal studies were randomized prospective cohort trials that compare non-protective CPPV or low tidal volume (LTV) with APRV [5, 49, 52, 54, 55, 57, 61, 62]. P-APRV was shown to reduce heterogeneity and both alveolar and alveolar duct micro-strain (i.e., change in alveolar size with applied stress) [54–56] suggesting a mechanism for the improvement in the efficacy experiments (Table 3) [5, 49, 57, 61, 62].

### Discussion

There has been no consensus on what parameters are essential to define a mechanical breath as being APRV, and thus, APRV settings have been inconsistent over the three decades since it was first described. However, no studies have shown that APRV is harmful or significantly inferior as compared with conventional mechanical ventilation. In 2005, Habashi clearly defined the settings for what we have termed personalized APRV or P-APRV [6]. P-APRV has a prolonged T_High_ and very brief T_Low_ duration, which is set by lung mechanics using the change in the slope of the expiratory flow curve [6]. Not only is the T_Low_ very brief in P-APRV, it is set by analyzing the slope of the expiratory flow curve and therefore adaptive to changes in the patient’s lung mechanics (Fig. 2b). Using these precisely controlled APRV settings, a number of studies have shown that P-APRV recruits and stabilizes the alveoli and alveolar ducts [54–56] and reduces the incidence of ARDS in multiple animal models [5, 57, 62]. A meta-analysis has shown a reduction in ARDS incidence in trauma patients [53]. Given this lack of consensus on how APRV should be set, the remainder of the “Discussion” will be divided into the results obtained from the F-APRV and P-APRV subgroups, rather than discuss the findings from each experiment with a different %CPAP, T_Low_, and/or P_High_ settings.

#### F-APRV studies

Since, as previously mentioned, most of the animal and human studies were crossover experiments (i.e., switching from CPPV or spontaneous breathing to F-APRV in the same animal or patient), the majority of published APRV studies do not address efficacy (i.e., is APRV superior to conventional ventilation). It is important to note that APRV did not cause a significant negative impact, as compared with CPPV or spontaneous breathing, on the physiologic parameters that were measured in any of the crossover studies (Table 1 and 3). In the randomized prospective animal studies, F-APRV was shown to be beneficial. It was shown that APRV reduced lung water and HMGB1 in rabbits [18], improved systemic and cerebrospinal blood flow in swine [16], improved oxygenation and attenuated ventilator-induced lung injury (VILI) in rabbits [26], and improved oxygenation and reduced lung injury in swine [7].

In humans, Putsenen et al. showed that APRV with spontaneous breathing increased oxygenation, cardiac index, and pulmonary compliance, with reduced sedative requirements compared with CPPV in humans [39]. Varpula et al. reported similar mortality and ventilator-free days for APRV and CPPV, and also demonstrated that it is feasible to utilize APRV with prone positioning. In that study, T_Low_ was set specifically to allow expiratory flow to reach zero, which would allow the lung to collapse during expiration [43, 44]. Maxwell et al. showed no difference between low tidal volume ventilation (LTV) and APRV regarding mortality, ventilator days, ICU length of stay, or complication rates despite increased baseline disease severity in the APRV group [38]. In human retrospective trials, the APRV methodology used was not consistent among the studies analyzed (Table 3). Despite these inconsistencies, Gonzalez et al. showed that APRV reduced peak inspiratory pressure (PIP) while maintaining similar oxygenation levels [32]. Hanna et al. showed increased PaO_2_/FiO_2_ (P/F) ratio, lung procurement rate, and graft survival when used in organ donors where the lungs were transplanted [33].

A retrospective study examining trauma patients by Maung et al. demonstrated increased ventilator days on APRV compared with an unspecified method of CPPV [47]. However, this study excluded all mortality in the interest of investigating weaning and the baseline characteristics of the groups were different, with more severe chest trauma and decreased P/F ratio at the start in the APRV group. Further, this study used respiratory therapist-guided protocol weaning only in the CPPV group, whereas the APRV group had random, non-protocolized weaning based on physician guidance.

#### P-APRV

Despite defining the role of tidal volume (Vt) and plateau pressure on VILI in ARDS patients [63], our current understanding of the parameters comprising the mechanical breath that either propagate or impede progressive acute lung injury (ALI) is incomplete. The mechanism of VILI is believed to be the evolution from a normal homogenously ventilated lung into a heterogeneously ventilated lung, with collapse and edema-filled alveoli adjacent to open alveoli. This heterogeneity results in stress concentrators and recurrent alveolar collapse and reopening with each tidal breath that amplify lung tissue injury, instigated by the initial insult such as sepsis, trauma, or pneumonia [64–66]. Thus, the ventilation strategy that restores or maintains homogeneity would minimize VILI and obstruct progressive ALI [64, 66, 67].

P-APRV uses a 90 % CPAP phase that recruits alveoli resulting in homogeneous lung inflation (Fig. 2a) and a brief release phase with the T_Low_ personalized to the mechanics of the lung (Fig. 2b) producing a nearly static ventilated lung, which prevents alveolar collapse and reopening, thereby reducing dynamic tissue strain [54, 56]. The question is, do these APRV settings that stabilize alveoli protect the lung?

In prospective outcome animal experiments, P-APRV resulted in an increase in oxygenation along with a decrease in histopathologic injury as compared with CPPV and LTV ventilation strategies [5, 52, 57, 61, 62]. Several studies showed that P-APRV resulted in an increase in surfactant protein concentration [5, 57, 62], while two showed a reduction of inflammatory markers (Table 2) [49, 57].

Kollisch-Singule et al. [54–56] conducted three micro-anatomic studies (i.e., alveoli and alveolar ducts) that demonstrated reduced alveolar and conducting airway micro-strain as well as increased alveolar homogeneity using P-APRV in which the T_Low_ was set to maintain an EEF/PEF ratio 75 or 10 %. The T_Low_ set using an EEF/PEF ratio of 75 % was sufficiently short to stabilize alveoli and prevent alveolar collapse, whereas extending the T_Low_ (EEF/PEF ratio 10 %) resulted in alveolar collapse and instability. These studies add mechanistic support to efficacy studies showing that preemptive P-APRV reduced ARDS incidence in a clinically applicable porcine ARDS model [5, 57].

Davies et al. [52] showed increased oxygenation with P-APRV as compared with LTV, with no significant differences in cerebral ischemia in a swine model of concomitant brain and lung injury (Table 2). In patients, Yoshida et al. [60] demonstrated increased oxygenation and mean arterial pressure (MAP) with decreased atelectasis using P-APRV as compared to LTV ventilation. Walsh et al. [58] showed improved pulmonary blood flow when using P-APRV versus CPPV ventilation post-operatively after tetratology of Fallot repair or cavopulmonary shunt in neonates (Table 4).

Furthermore, Andrews et al. [50] in a meta-analysis showed a tenfold decrease in ARDS incidence as well as a threefold decrease in mortality when compared to trauma patients with similar injuries that were treated with standard of care ventilation in 15 trauma intensive care units (Table 4). However, all of the human trials testing the P-APRV method [50, 53, 58–60] are retrospective studies, and as such, it is not clear that the precise P-APRV settings were followed, aside from the study by Andrews et al. [50] that strictly adhered to the P-APRV protocol throughout the study in trauma patients. Despite this, the human results are in concordance with multiple animal studies [5, 49, 54–57, 61, 62] that support the clinical data [50] that P-APRV may be used to reduce the incidence of acute respiratory distress syndrome (ARDS) more effectively than conventional LTV ventilation applied early or after the onset of ARDS.

#### A note on mechanical ventilators

An often under-analyzed aspect of mechanical breath delivery is the ventilator used to deliver the breath. Every ventilator has a specific design with differing resistances built into the ventilator gas path, responses to spontaneous ventilation, as well as software quirks that affect breath delivery. APRV may be named in various ways based on the ventilator in use such as (1) APRV (Drӓger Evita, Savina and V series, Hamilton G5), (2) Bi-Vent (Maquet Servo-i), (3) BiLevel (Engström Carestation, Puritan Bennett 840 & 980), and (4) APRV/Biphasic (Viasys Avea). However, even more problematic than the different names is the wide variation in their implementation of APRV.

In particular, the ability to control key APRV parameters such as the T_Low_ varies, and fine control of this parameter is critical to properly set P-APRV (Fig. 2b). APRV strategies on some ventilators have deviated from the original concept of a timed CPAP with a release phase, by adding pressure support (PS). This requires a trigger that creates a need for synchronization with the ventilator resulting in automated changes to both the inspiratory and expiratory duration. More specifically, the T_Low_, which is critical to control end-expiratory lung volume and prevent airway closure, spontaneously adjusts when PS is added to this mode on these ventilators, regardless of the T_Low_ setting, producing large and variable tidal volumes leading to lung volume loss and alveolar instability. So, even if APRV was set accurately by the clinician, the ventilator may automatically adjust the duration of the release phase (T_Low_) ultimately allowing the lung to collapse. This effect would be similar to conventional ventilation automatically adjusting Vt or PEEP without physician input.

Furthermore, many researchers define APRV as “extreme” inverse-ratio pressure-control ventilation (IR-PCV). However, unlike IR-PCV, P-APRV does not set a defined I:E ratio. Rather, the CPAP phase is briefly interrupted by a “release phase” (i.e., T_Low_) that is established by independently setting a T_Low_ based upon analysis of the expiratory flow curve. Further, patients can breathe spontaneously throughout the entire respiratory cycle in APRV, superseding the set I:E ratio. Unfortunately, many times the exact make and model of the ventilator was not reported in the papers reviewed, and thus, we could not categorize studies by the ventilator used, but it is an important factor to consider if the ventilator being used can accurately deliver P-APRV.

#### Current state of APRV and clinical implications

As described previously, there is a paucity of data testing APRV with identical settings, especially in clinical trials almost all of which were retrospective or crossover studies. Rose et al. [68] reviewed the literature in 2008 and concluded that there is a lack of consistency in APRV settings making comparison with conventional ventilator strategies difficult. Another recent review by Facchin et al. [69] examined the current literature for both APRV and high-frequency oscillatory ventilation (HFOV) for treatment and prevention of ARDS; the authors concluded that there is inconsistent evidence and a lack of high quality trials to make conclusions regarding APRV or HFOV efficacy.

It is clear from the studies examined in this review that APRV has evolved from a mechanical breath defined as a prolonged CPAP phase with a brief release phase [1] into a highly sophisticated, dynamic mechanical breath with precise settings that are responsive to changes in lung physiology (i.e., P-APRV) [6]. Although the mechanical breath used by both Downs and Habashi are referred to as APRV, the actual mechanical breath created by each is very different (Fig. 1a, d) [2, 57] as is the breath created and studied in other experiments [3, 9] (Fig. 1b, c). The current use of the APRV acronym remains an imprecise term that demands a specific definition of parameter settings to be utilized consistently. Obviously, some APRV settings are going to superior to others in their ability to protect the lung.

P-APRV appears to be an exciting and novel open lung strategy that may significantly reduce ARDS incidence, morbidity, and mortality of established ARDS. Data suggests that rather than overdistending alveoli, the extended T_High_/P_High_ redistributes gas from the alveolar ducts to the alveoli, where it belongs [54, 55] and changes heterogeneous to homogeneous alveolar ventilation [56]. The calculated strain on alveoli was significantly reduced demonstrating that mechanical stretch on the alveolar wall was decreased. These data have led us to conclude that the extended inspiratory duration has a powerful positive impact on reducing strain at the alveolar level. We hypothesize that the mechanism of this protection (i.e., reduce alveolar strain) is secondary to increased lung volume and the number of recruited alveoli. The extended T_High_ “nudges” open alveoli over a several hours in a non-pathologic manner resulting in open lung ventilation. Since the lung becomes fully recruited with significantly improved compliance, even with relatively high tidal volumes (10–14 cm^3^/kg), the driving pressure is not increased (unpublished observations).

## Conclusions

Although many settings have been used, none of the studies reviewed showed a worse outcome using APRV as compared with CPPV, with many studies showing significant benefits in cardiopulmonary variables. The evolution of APRV methodology has been drastic from Downs and Stock (F-APRV) [1] to the P-APRV method of Habashi [6]. P-APRV allows for a personalized control of lung stability on a breath-to-breath basis that is not possible with other modes of ventilation. P-APRV is an adaptive, flow directed, duration dependent ventilation strategy that adapts the setting to each patient regardless of their lung pathophysiology. This personalized, adaptive mechanical breath may prove more efficacious at treating and preventing ARDS than the current standard of care. Ultimately, more studies are needed using consistent and well-defined settings to identify the optimal APRV breath necessary to maximize lung protection.

## Abbreviations

ALI: acute lung injury
APRV: airway pressure release ventilation
ARDS: acute respiratory distress syndrome
BiPAP: biphasic positive airway pressure
CPAP: continuous positive airway pressure
CPPV: conventional positive pressure ventilation
F-APRV: fixed-setting airway pressure release ventilation
HFOV: high frequency oscillatory ventilation
LTV: low tidal volume
MAP: mean arterial pressure
P/F: PaO_2_/FiO_2_
P-APRV: personalized airway pressure release ventilation
P_aw_: airway pressure
PCO_2_: partial pressure of carbon dioxide
PEEP: positive end-expiratory pressure
PEF: peak expiratory flow
P_High_: pressure during CPAP phase
PIP: peak inspiratory pressure
P_Low_: pressure during release phase
PS: pressure support
PSV: pressure support ventilation
SB: spontaneous breathing
T_High_: time at CPAP/P_High_
T_Low_: time at release/P_Low_
VILI: ventilator-induced lung injury
Vt: tidal volume
